# How Controlled is the Expansion of VIATORR CX?

**DOI:** 10.1007/s00270-023-03383-4

**Published:** 2023-02-24

**Authors:** Tatjana Dell, Matthias Menne, Julia Wagenpfeil, Michael Praktiknjo, Christian Jansen, Alexander Isaak, Narine Mesropyan, Ulrich Steinseifer, Ulrike Attenberger, Julian Luetkens, Carsten Meyer, Daniel Kuetting

**Affiliations:** 1 Department of Diagnostic and Interventional Radiology and Quantitative Imaging Lab Bonn (QILaB), University Hospital Bonn, Venusberg-Campus 1, 53127 Bonn, Germany; 2 Department of Cardiovascular Engineering, Institute of Applied Medical Engineering, Helmholtz Institute Aachen, RWTH Aachen University, Pauwelsstraße 20, 52074 Aachen, Germany; 3 Department of Internal Medicine I, Center for Cirrhosis and Portal Hypertension Bonn (CCB), University Hospital Bonn, Venusberg-Campus 1, 53127 Bonn, Germany

**Keywords:** Transjugular intrahepatic portosystemic shunt (TIPS), Physical properties, Hepatic encephalopathy, Portosystemic over-shunting, In vitro mechanical test, Gore VIATORR-Controlled Expansion Endoprosthesis (VCX)

## Abstract

**Purpose:**

To investigate and compare the physical properties of the new generation Gore VIATORR-Controlled Expansion Endoprosthesis (VCX) to those of the predecessor VIATORR stent in an in vitro experimental setup.

**Materials and Methods:**

A total of 12 stents (8 VCX; 4 VIATORR; GORE, USA) were examined. Radial resistive force (RRF) and chronic outward force (COF) were assessed using a radial force testing machine (RX-650, Machine Solutions Inc., USA). To assess the radial forces of the VCX above 8 mm, balloon expansion was performed between cycles.

**Results:**

All VCX stents show an abrupt decrease in COF at an external diameter of 8.3 mm; RRF decreases likewise at an external diameter of 8.5 mm. The predecessor VIATORR stent without the “controlled expansion” feature shows linear radial force reduction until full expansion at a diameter of 10 mm.

The physical properties of the VCX can be altered by balloon modulation. Point of COF (RRF) reduction shifts to 8.5 mm (8.6 mm), 8.6 mm (8.8 mm) and 9.3 mm (9.6 mm) following modulation with a 8 mm, 9 mm and 10 mm balloon.

**Conclusions:**

The VCX shows an abrupt and disproportionate decrease in COF and RRF at an external diameter of 8.3 mm, thus passive expansion to its nominal diameter of 10 mm is not to be expected. By means of balloon dilatation the physical properties of the stent can be altered, enabling customized TIPS creation. The previous VIATORR stent shows continuous COF and RRF until total expansion.

## Introduction

Transjugular intrahepatic portosystemic shunt (TIPS) is now considered the procedure of choice for the treatment of portal hypertension-related complications, especially variceal bleeding and refractory ascites in selected patients [1]. Currently, TIPS creation is most commonly performed with the VIATORR stent (W.L. Gore & Associates Inc., Flagstaff, AZ, USA) [2]. As TIPS creation may lead to complications such as deterioration of liver function, cardiac decompensation and hepatic encephalopathy (HE), especially if shunt fraction is too high, efforts have been made to develop techniques to balance the desired therapeutic effect while minimizing the risk of over-shunting [3]. To facilitate these efforts, the design of the VIATORR stent was updated (now VCX stent), in theory allowing for controlled calibration of portosystemic gradients during TIPS creation without post-interventional gradual passive stent expansion [4].

As data regarding the expansion properties of the VIATORR stent is currently lacking, the rationale of this study was to gain more detailed understanding on the physical properties of the VCX stent in comparison to the previous VIATORR stent.

## Materials and Methods

The study was designed by an interventional radiologist with 10 years of experience in the management of portal hypertension and by a medical engineer with research focus on cardiovascular technology and in vitro testing of medical devices.

**Fig. 1:**
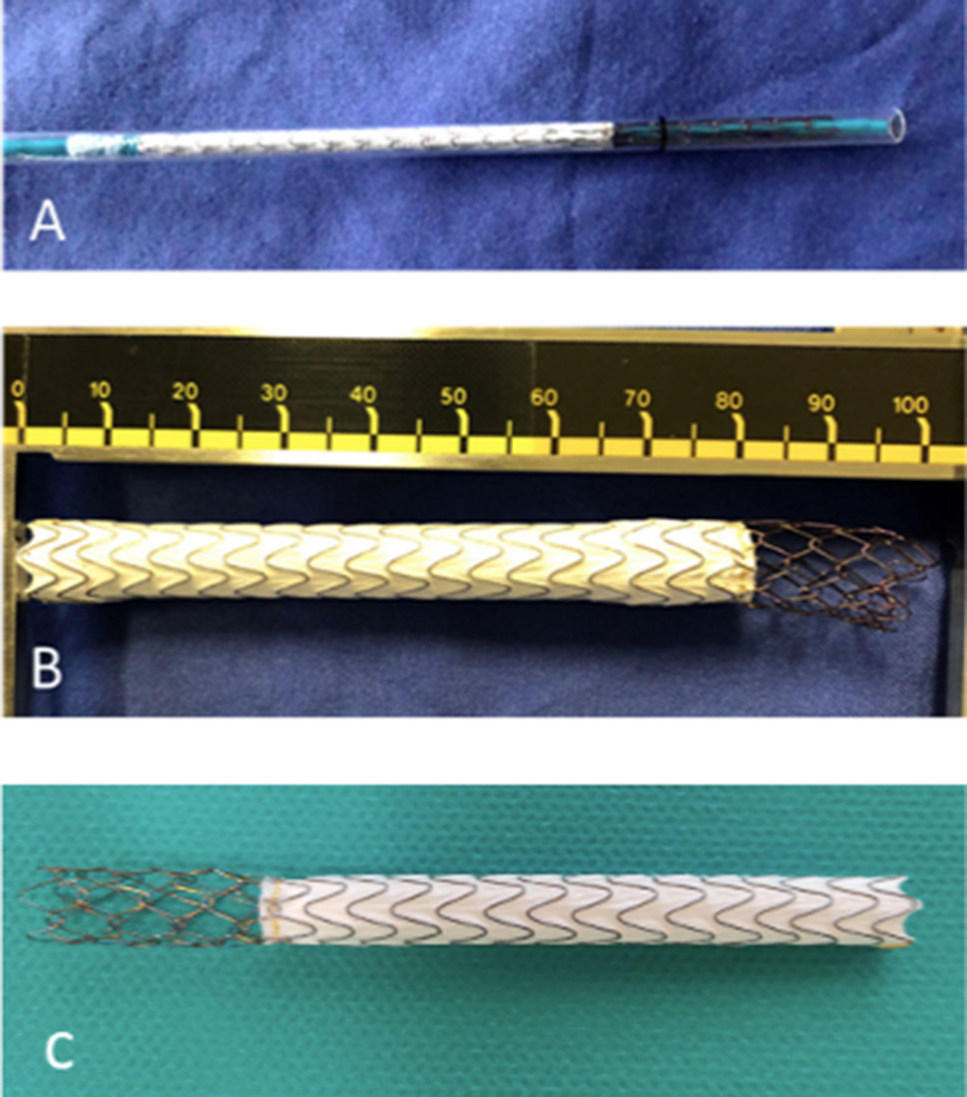
Image of the tested stents: VIATORR-controlled expansion stent (VCX) inside its catheter (**A**) and fully released (**B**). Predecessor VIATORR stent (W.L. Gore & Associates Inc., Flagstaff, AZ, USA) as a reference (**C**)

Twelve stents were investigated: eight VIATORR-controlled expansion stents (VCX), and four predecessor VIATORR stents as a reference (Fig. 1).

Five of the VCX were 100 mm long with an 8 cm covered portion, while the remaining three VCX and the four reference stents were 80 mm long with a 6 cm covered segment. To allow for comparability of the results despite the different lengths of the stents measured forces were normalized to 1 cm of length.

**Fig. 2:**
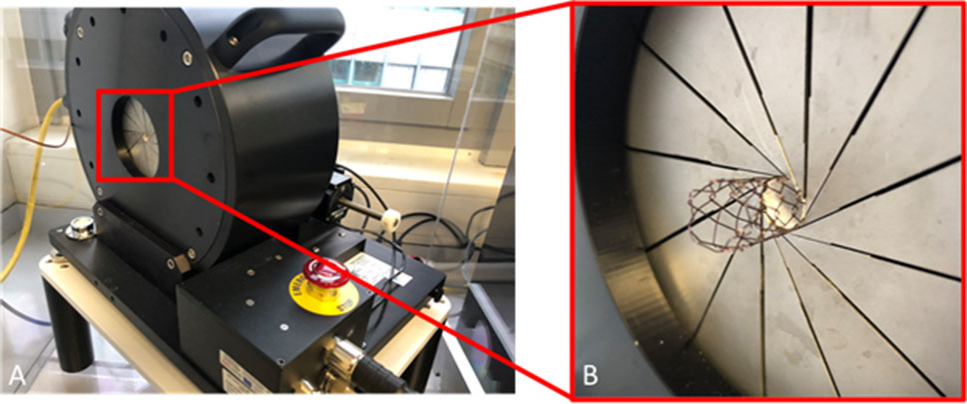
Radial force testing machine: **A** RX-650 (Machine Solutions Inc., Flagstaff, AZ, USA) with Stent, **B** close up of crimping jaw with stent inside

**Fig. 3:**
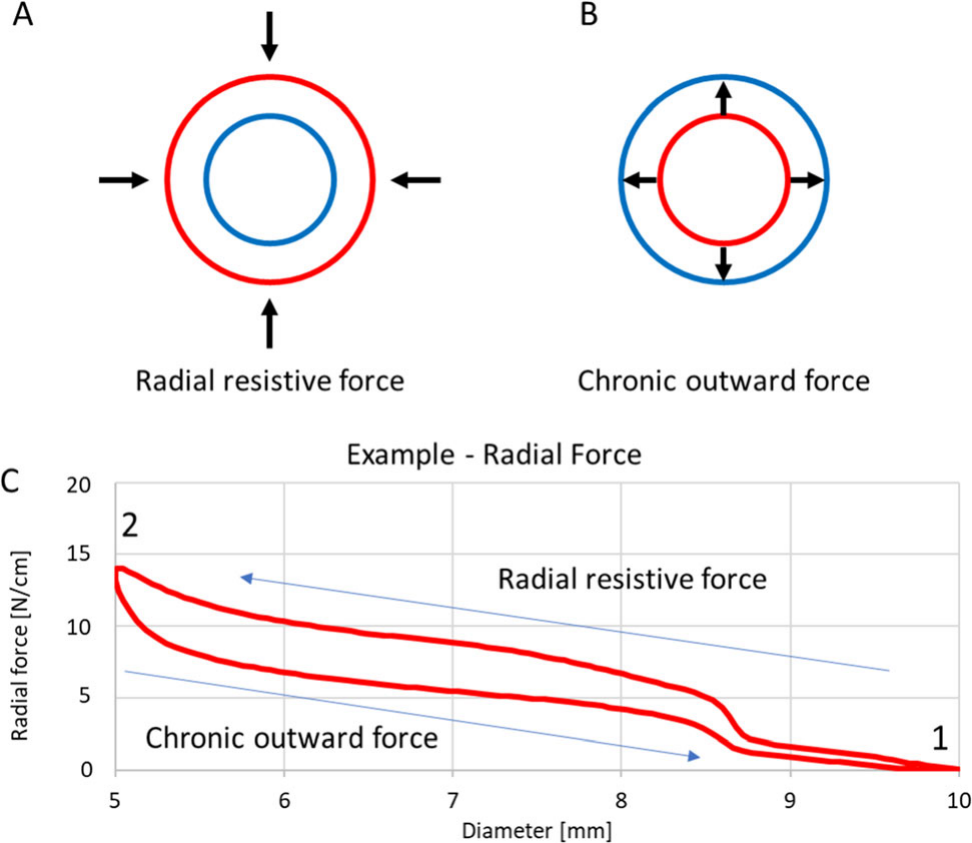
Schematic drawing of circumferential stent compression during crimping (**A)** and stent self-expansion (**B)** performed on a radial force tester. Schematic drawing of deformation characteristics of a self-expanding (nitinol) stent during crimping and expansion (**C**). The test starts fully expanded at 10 mm (1). The stent is then crimped to a nominal outer diameter of 5 mm (2) and self-expands back to the initial 10 mm. The force exerted during crimping is the radial resistive force, the force exerted during self-expansion is the chronic outward force

Radial resistive force (RRF) and chronic outward force (COF) were measured using a RX-650 radial force tester (Machine Solutions Inc., Flagstaff, AZ, USA) (Fig. 2), as previously described [5, 6]. The RRF is the force with which the stent withstands crimping from the outside. The COF is a measure of the force a stent exerts as it expands to its nominal diameter. The terms RRF and COF have been coined by Duerig and Stoeckel to better describe the specific characteristics of nitinol stents [7]. Both characteristics of radial force were analyzed using the radial force-tester method (Fig. 3) [8]. In this study, the covered segments of examined stents were inserted into the tester’s crimping jaw still covered by the protective sheath in a crimped state. The sheath was then removed and the deployment line was pulled allowing the stent to gradually expand in the crimping jaw (expansion rate of 0.5 mm/s). The stent expands passively, exerting an outward force on the measuring blades. The force measured during self-expansion is the COF. After full expansion, the stents were crimped down to 5 mm by the crimping jaw at a rate of 0.5 mm/s. The force measured during crimping is the RRF. The stents were then balloon expanded to 8, 9, and 10 mm, respectively, using a non-compliant balloon (Mustang Boston Scientific Corp., Marlborough, MA, USA) inflated to its respective nominal pressure. The balloon expansion was performed outside of the radial force tester. After each balloon expansion, the stents were inserted into the radial force tester, crimped down to 5 mm and then allowed to expand to their full diameter by themselves to investigate the impact of balloon expansion on COF and RRF.

Testing was performed inside a heating chamber set to 37 ± 1 °C to simulate in vivo conditions. Measured radial force and diameter values are reported as mean ± standard deviation. The reported values are means of the measurements of all stents. Diameter values refer to the outer diameter of the stents. Accuracy, resolution and repeatability of the RX-650 radial force tester according to the manufacturer are: diameter accuracy: 0.2%; diameter resolution: 0.01 mm; radial force repeatability: 1%; radial force resolution: 0.06%.

## Results

**Fig. 4:**
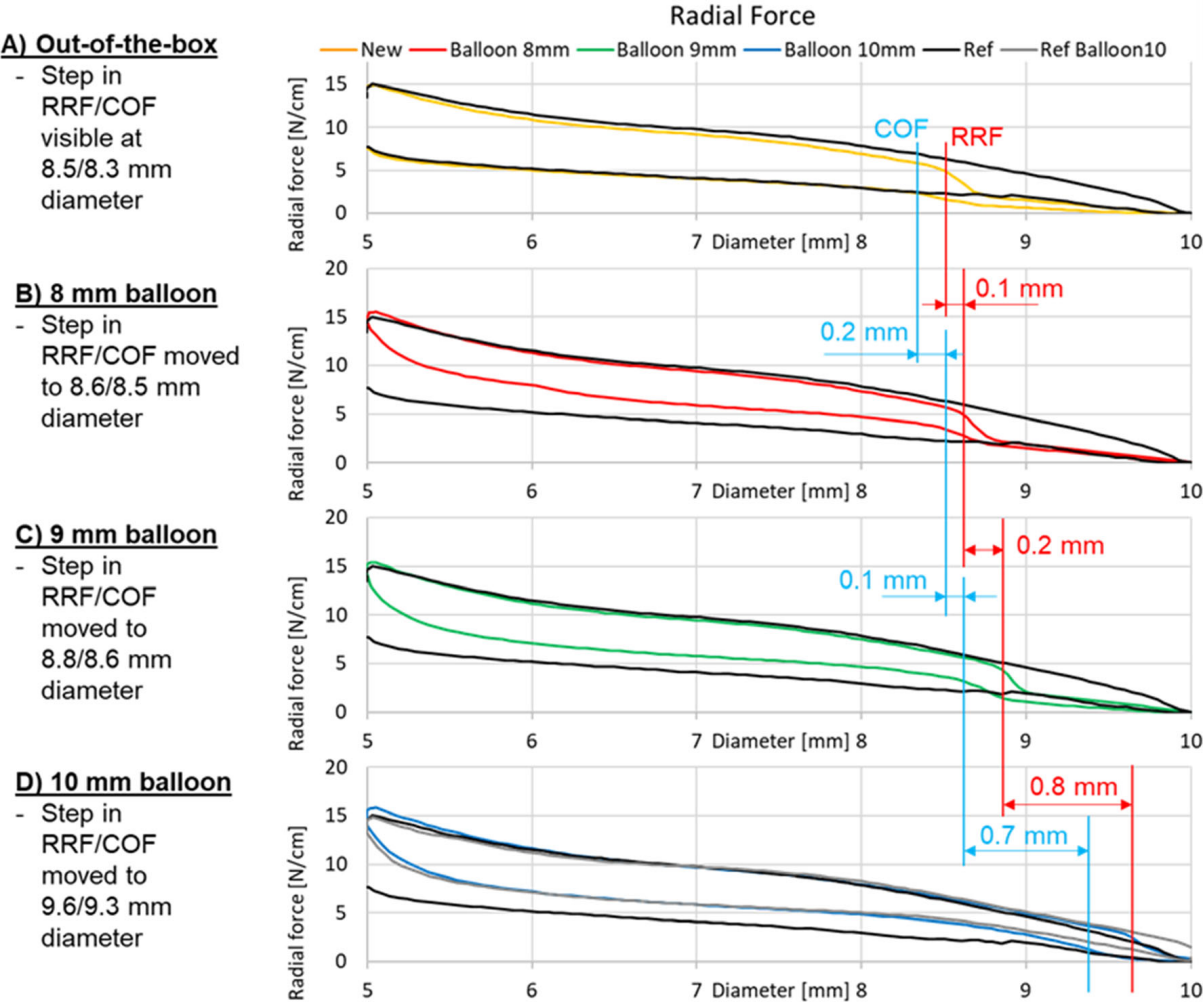
Results of the radial force measurements for the different stents. The predecessor VIATORR stent expanded completely to an outer diameter of 10 mm (black line). For the new generation devices (yellow line) a step in resistive radial force is visible at 8.47 ± 0.06 mm diameter at out-of-the-box condition. This step was moved toward 8.56 ± 0.01 mm, 8.81 ± 0.04 mm and 9.62 ± 0.06 mm diameter after inflation with an 8 mm (red line), 9 mm (green line) and 10 mm balloon (blue line), respectively

Detailed results of the radial force-tester method are given in Fig. 4a–d. All VCX stents of the new generation (out-of-the-box conditions) show an abrupt reduction in RRF of around 50% at an outer diameter of 8.47 ± 0.06 mm (Fig. 4a, upper yellow line). COF showed disproportionate decrease at an outer stent diameter of 8.31 ± 0.06 mm.

**Fig. 5:**
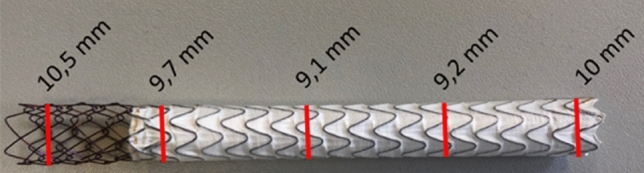
At 37 °C out of the box the VCX did not expand uniformly to the same size and took on a dogbone shape: it expanded to 9.1 mm in the center and to 9.7 mm at the edges. The uncovered edge of the stent even expanded to almost 10.5 mm

In comparison COF and RRF curves of the predecessor VIATORR stent (Fig. 4a; black line) revealed no disproportionate reduction in force during expansion or compression. The predecessor VIATORR stent expanded completely to an outer diameter of 10.40 ± 0.70 mm. At 37 °C out of the box the VCX did not expand uniformly to the same size and took on a dogbone shape: it expanded to 8.85 ± 0.33 mm at the narrowest point and to a maximum of 9.61 ± 0.47 mm in the covered part. The narrowest point was in the covered segment of the stent close to the middle at 52.83 ± 1.85% of the stent’s length measured from the uncovered portion toward the covered portion. The uncovered edge of the stent expanded to almost 10.20 ± 0.52 mm. One example is shown in Fig. 5.

After dilatation of the VCX with an 8 mm balloon the point of RRF reduction shifted from 8.47 ± 0.06 mm to 8.56 ± 0.01 mm. Following expansion with a 9 mm balloon RRF reduction was noted at 8.81 ± 0.04 mm; following 10 mm dilatation at 9.62 ± 0.06 mm.

Likewise, the diameter at which COF declined extensively changed following balloon dilatation. After dilatation of the VCX with an 8 mm balloon marked COF reduction occurred at 8.47 ± 0.01 mm, instead of originally 8.31 ± 0.06 mm. Following expansion with a 9 mm marked COF reduction was noted at 8.63 ± 0.04 mm; following 10 mm dilatation at 9.32 ± 0.03 mm.

The largest change in COF/RRF was noted following modulation with a 10 mm balloon. In contrast to RRF, where balloon expansion merely lead to a shift of the force curve, COF values increased in total following balloon modulation (Fig. 4b–d, lower red/green/blue lines).

The previous version of the VIATORR stent only showed a change in radial forces following modulation with a 10 mm balloon. Balloon expansion led to an elevation of COF values as well as to a slight increase in RRF between diameters of 8 and 10 mm (Fig. 4d, gray line).

## Discussion

The main findings of this study are that the updated design of the VIATORR stent including a restrictive PTFE sleeve prevents unwanted complete passive expansion.

Despite continued refinements in technique and devices, hepatic encephalopathy (HE) remains a major drawback of TIPS with a reported incidence of 20% within the first year following intervention [9, 10]. Apart from liver function, the degree of portosystemic gradient reduction has an impact on the incidence of HE [11, 12]. In this regard, it has been shown that following TIPS placement the magnitude of the portosystemic gradient negatively correlates with the shunt diameter of the placed stent [13]. Therefore, attempts have been made to regulate shunting by means of shunt diameter adaptation. The use of small-diameter (6–8 mm) stents has been advocated for the prevention of HE [14] but showed inconsistent results in terms of efficacy for controlling complications of portal hypertension [15]. Implantation of a large-diameter (i.e., 10 mm) subtotally expanded stent (6–8 mm) has been proposed as an alternative approach [13, 14]. In theory this approach allows for gradient adapted TIPS creation with the prospect of sequential balloon-assisted shunt expansion in cases of insufficient clinical response. However, the success of this approach is dependent on the physical properties of the implanted stent.

The current results further prove that the previous generation VIATORR has the tendency to expand completely. The results are in-line with previous studies that investigated VIATORR configuration in in vivo settings and found that the stent tends to passively expand to its nominal diameter following subtotal initial dilatation [2, 3, 15–17], thus potentially leading to excessive portosystemic gradient reduction. The duration of passive expansion was neither unanimous nor predictable in the various studies.

**Fig. 6:**
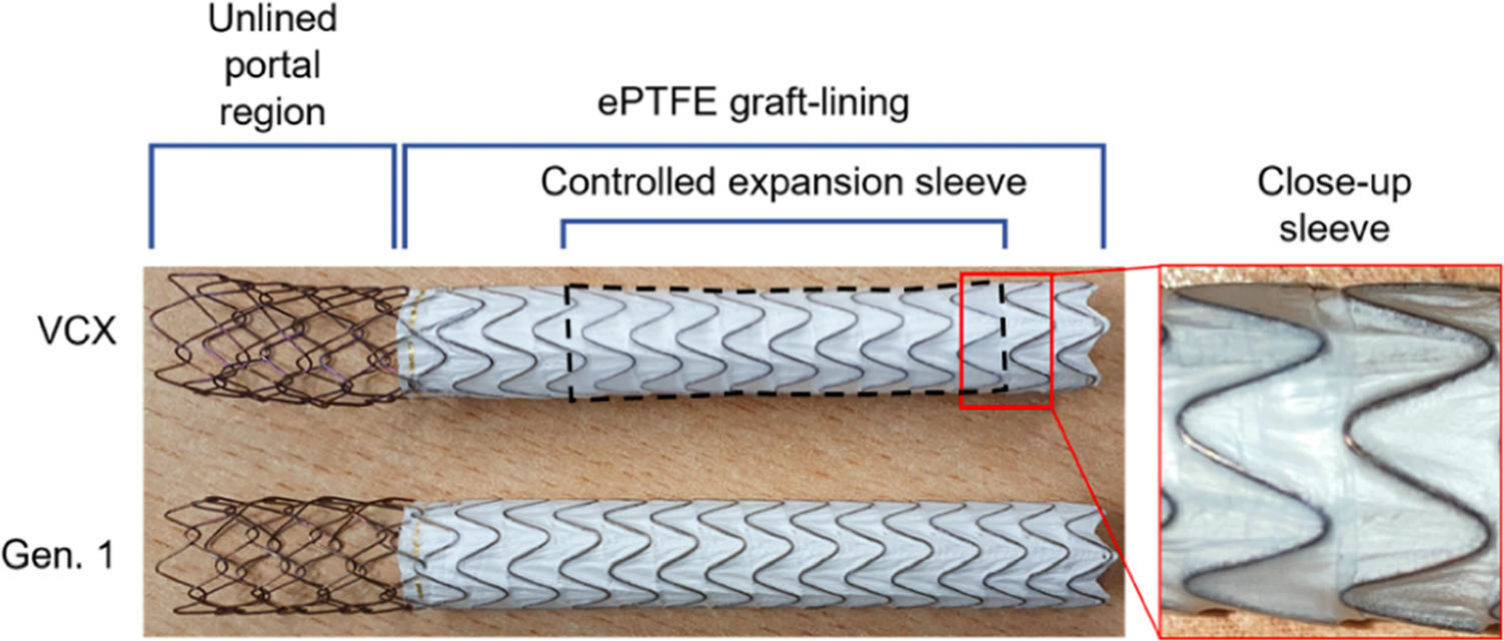
Design of VIATORR-Controlled Expansion Endoprosthesis (VCX) and predecessor VIATORR stent (Gen. 1, W.L. Gore & Associates Inc., Flagstaff, AZ, USA). The new feature of controlled expansion sleeve guarantees the size of the diameter during implantation

As a result, an updated version of the VIATORR stent was designed with the goal of controlled expansion. The predecessor VIATORR stent is a nitinol-based stent with an uncovered 2-cm-long self-expanding chain-linked portion and an ePTFE-covered spiral nitinol portion, constrained by a suture. The VIATORR CX (VCX) is similar in design to the original VIATORR with an additional outer constraining balloon-expandable sleeve on the lined region of the stent graft. This lined region was added to allow for controlled balloon-assisted adjustment of the stent diameter in a range between 8 and 10 mm (inner diameter) (Fig. 6).

In TIPS creation COF is of higher interest, as customized gradient adaptation can only be achieved if surplus passive expansion is not an issue. A significant reduction in COF was found in all VCX at an outer stent diameter of 8.31 ± 0.06 mm to values below the previous VIATORR version. This effect is reinforced by a 50% RRF reduction, which occurs at an outer diameter of 8.47 ± 0.06 mm. Thus, it can be assumed that after implantation the VCX will open to a maximum diameter between 8.3 and 8.5 mm without subsequent balloon modulation in most cases. In vivo the duration and extent of passive expansion depends on numerous factors (i.e., degree of liver stiffness, and diameter of pre-dilatation), thus the final diameter of the VCX is difficult to predict if post-dilatation is not performed.

After modulation with an 8 mm balloon the stent expands to a maximum diameter between 8.47 ± 0.01 and 8.56 ± 0.01 mm. Following modulation of the VCX with a 9 mm balloon only slightly alters the physical properties and at most could prevent some recoil in case of increased liver stiffness. To the largest degree, the radial forces change after a 10 mm balloon modulation: a decrease in COF is observed at 9.32 ± 0.03 mm, and a decrease in RRF at 9.62 ± 0.06 mm. Stent recoil was seen after balloon modulation of the VCX with the 9 and 10 mm balloon, respectively. The recoil was most pronounced after modulation with the 10 mm balloon. Clinicians should bear these findings of mechanical behavior of the VCX stent in mind when performing TIPS creation with stent placement and possible balloon dilatation.

The current study is limited by the experimental character. Although examinations were performed simulating in vivo conditions, it remains unclear whether results are transferable to in situ use. COF and RRF were measured after re-crimping; a process which typically does not take place in vivo. In addition, since older generation VIATORR stents are no longer available, the overall significance of comparing the two stents is limited. However, as the older generation VIATORR was a commonly used and clinically well-known stent, understanding of how the new VCX compares to the previous VIATORR version in vitro, may help clinicians to better predict its behavior in situ. More relevant would also be the comparison between VCX and the balloon-expandable stents recently used instead of VIATORR [18, 19]. However, this is not possible because the test method used is only suitable for nitinol stents. Finally, this is a study of low sample size that also prohibits any statistical calculations.

## Conclusion

The results of this study demonstrate that the previous generation VIATORR stent has the tendency to expand completely to its nominal diameter and thus, is not equipped with the physical properties necessary for durable subtotal dilatation. The physical properties of the VCX can be modulated by balloon modulation. The design of the new VCX with a balloon-expandable sleeve, ensures diameter stability, thus allowing for individualized calibration of the portosystemic gradient.

## Abbreviations

VCX: VIATORR-Controlled Expansion Endoprosthesis
RRF: Radial resistive force
COF: Chronic outward force
TIPS: Transjugular intrahepatic portosystemic shunt
HE: Hepatic encephalopathy
